# Recent Advances in the Development of Peptide Vaccines and Their Delivery Systems against Group A Streptococcus

**DOI:** 10.3390/vaccines7030058

**Published:** 2019-07-01

**Authors:** Armira Azuar, Wanli Jin, Saori Mukaida, Waleed M. Hussein, Istvan Toth, Mariusz Skwarczynski

**Affiliations:** 1 School of Chemistry and Molecular Biosciences, The University of Queensland, St. Lucia, QLD 4072, Australia; 2 Pharmaceutical Organic Chemistry Department, Faculty of Pharmacy, Helwan University, Helwan, Cairo 11795, Egypt; 3 School of Pharmacy, Woolloongabba, The University of Queensland, St. Lucia, QLD 4072, Australia; 4 Institute of Molecular Bioscience, The University of Queensland, St. Lucia, QLD 4072, Australia

**Keywords:** peptide-based subunit vaccine, group A Streptococcus antigens, adjuvant, delivery system, M protein

## Abstract

Group A Streptococcus (GAS) infection can cause a variety of diseases in humans, ranging from common sore throats and skin infections, to more invasive diseases and life-threatening post-infectious diseases, such as rheumatic fever and rheumatic heart disease. Although research has been ongoing since 1923, vaccines against GAS are still not available to the public. Traditional approaches taken to develop vaccines for GAS failed due to poor efficacy and safety. Fortunately, headway has been made and modern subunit vaccines that administer minimal bacterial components provide an opportunity to finally overcome previous hurdles in GAS vaccine development. This review details the major antigens and strategies used for GAS vaccine development. The combination of antigen selection, peptide epitope modification and delivery systems have resulted in the discovery of promising peptide vaccines against GAS; these are currently in preclinical and clinical studies.

## 1. Introduction

Group A Streptococcus (GAS)

Group A Streptococcus (*Streptococcus pyogenes*, or GAS) is a Gram-positive, pathogenic bacterium that exclusively infects humans [1,2]. This bacterium causes a vast array of diseases, ranging from non-invasive infections to invasive and post-infectious diseases (Table 1). GAS resides on the surface of the skin or throat, which act as major entry sites for infection [1,2,3]. Streptococcal pharyngitis, also known as “strep throat”, is the most common infection caused by GAS colonisation of the throat [2,3]. Although it is a benign infection, recurrent or severe cases of streptococcal pharyngitis can lead to the development of life-threatening diseases, such as rheumatic fever (RF) and rheumatic heart disease (RHD) [3]. These autoimmune disorders are presumably triggered by production of antibodies against GAS that also recognize (a) human cardiac myosin due to its sequence homology with GAS antigens [4,5,6], and (b) collagen due to the binding of certain types of GAS with human collagen IV [6,7,8].

The World Health Organization (WHO) has estimated that more than 100 million people worldwide suffer from GAS-related diseases, resulting in more than half a million deaths each year [12]. In the United States alone, 25 to 35 million cases of GAS infection are diagnosed each year, reflecting an annual health care cost of up to US$ 2 billion [13]. RHD, a life-threating post-infectious sequela of RF, affects over 33 million individuals worldwide and has resulted in approximately 350,000 premature deaths, according to a recently published report [14]. Nonetheless, these numbers are considerably underestimated due to the sparse data on fatal and nonfatal cases of RHD in developing countries. Based on a recent study of economically disadvantaged populations, the prevalence of RHD exceeded WHO’s predicted rates by a factor of 4 to 5 [15]. This estimate suggested that there are 62 to 78 million people worldwide who suffer from RHD, and an estimated 1.4 million deaths per year.

Due to the devastating effects of GAS on the human population, this bacterium has been listed as one of the top 10 pathogens with high global morbidity and mortality [5,16]. Despite the great need for an effective cure, there are still no GAS vaccines available on the market [17] and patients have to rely predominantly on antimicrobial therapy (penicillin, erythromycin or clindamycin), adjunctive treatment (intravenous immunoglobulin (IVIG)), and prophylactic measures [17,18,19]. GAS remains susceptible to antibiotics; however, antibiotic therapy is inadequate as a primary treatment for RF and RHD, where its only decreases the duration of illness and severity of the symptoms. Moreover, the increase in the clinical use of antibiotics has resulted in antimicrobial resistance among GAS [19,20,21], which further complicates the situation.

Limited access to healthcare and poor infection control are major contributors to the spread of GAS in economically disadvantaged populations [12,17,22]. These populations are more susceptible to RHD because initial infections are often either undetected or untreated. Epidemics of GAS diseases not only occur in developing countries, but they also affect indigenous populations within developed countries. Incidence rates of RF in Australian Aboriginal populations, particularly in the Northern Territory and north Queensland, are among the highest in the world [2]. A broadly protective GAS vaccine would provide a cost-effective strategy for preventing RF and RHD in some of the world’s most at-risk populations.

This article reviews major GAS antigens and approaches to develop effective and safe GAS vaccines. Special attention has been given to peptide-based subunit vaccines and their delivery strategies.

## 2. Vaccine Development against GAS

### 2.1. Vaccination

Vaccination is a public health intervention used to stimulate protective immunity against infectious diseases [23,24]. Traditional vaccines are made from whole attenuated or inactivated microorganisms that induce strong and long-lasting immune responses. Regardless of their high immunogenicity, the major drawback of traditional approaches is the presence of immunologically redundant components or biological impurities in the vaccines, which have the potential to induce allergic or even autoimmune responses in humans. Attenuation is not always sufficient to ensure the safety of vaccinations, as processed pathogens can return to their active form [24]. The production and distribution of traditional vaccines may also be limited by pathogens’ requirements for special conditions during culturing, storage, and transportation. To overcome the drawbacks of traditional vaccines, subunit vaccines that contain only essential antigens derived from the pathogen have been developed [25,26]. Although isolated antigens are less likely to induce autoimmune or allergic responses, these antigens are also less immunogenic and they are not able to stimulate strong, long-lasting immunity against infections. Thus, in addition to the required antigen, complementary immunostimulants (adjuvants) are also needed to produce effective subunit vaccines [27,28].

#### Peptide-Based Subunit Vaccines

The use of peptides as antigens is a modern vaccine approach that uses minimal microbial components to stimulate adaptive immunity against a pathogen [26]. The use of peptides instead of whole organisms or proteins can completely remove the problems associated with allergic and autoimmune responses [25]. Peptide antigens are normally chemically synthesised, making their production customisable, simple, reproducible, fast, cost-effective and free from biological contaminations. Peptide vaccines are usually water-soluble, can be freeze-dried and are more stable in storage conditions. Highly conserved peptides, or a mixture of several epitopes, can be used to cover different pathogen subtypes [25,26]. Peptide B-cell epitopes can induce antibody-mediated (humoral) responses, while T-cell epitopes mediate cellular adaptive immunity against a desired pathogen. B-cell epitopes need to maintain their native protein conformation to induce the required humoral immunity [9,25,26]. Epitope conformation can be stabilised via modifications, such as sequence flanking, cyclisation or stapling. In addition, disease-specific or universal T-helper (Th) CD4+ epitope must also be present in vaccines to induce adaptive immunity and memory immune responses. However, peptides are poor immunogens, can lose their native conformation, are susceptible to enzymatic degradation, and are not consistently recognised by host populations [9,26,29]. Therefore, they require additional immune stimulants (adjuvant) or delivery systems that target antigen presenting cells (APCs), particularly dendritic cells (DCs) and macrophages, to enable the stimulation of B- and T-lymphocytes and induce the desired immunity [27].

### 2.2. GAS Vaccine Development

The history of GAS vaccine development can be traced back to the early 20th century, with first attempts dated to 1923 [30,31,32]. Early GAS vaccine trials, which were based on live attenuated or inactivated GAS, failed to deliver safe and efficient products. These vaccine candidates only offered limited coverage, protecting against a narrow range of GAS strains, and simultaneously stimulated autoimmune responses, allergies, and/or inflammation [33,34]. A large clinical trial in the 1940s documented that people vaccinated with inactivated GAS vaccine suffered from severe side-effects, without building any protective immunity against GAS infection [35]. When whole bacteria were replaced with M protein (the major virulence factor of GAS), bactericidal antibodies against GAS were produced by patients [36], but autoimmunity was also triggered, which resulted in RF among vaccinated children [37]. In consequence, the United States imposed a federal ban on GAS vaccine tests in humans (the ban was removed in 2006) [30,38]. It is worth mentioning that the M protein used for these studies was not fully purified and was most likely contaminated with other GAS components, therefore autoimmune responses triggered by the vaccine were not necessarily related to M protein [30]. Other clinical trials that used well-defined and highly-purified M protein did not show cross-reactivity complications [39,40,41,42]. However, a variety of experimental and computational studies have suggested that M proteins contain the cross-reactive B and T cell epitopes with human tissues [5,43,44,45,46,47,48,49,50]. While single vaccination with M protein may not generate significant immune responses against cross-reactive epitopes in the general population, individuals with pre-existing exposure to GAS may develop autoimmune reactions. Therefore, current vaccine constructs do not use whole M protein, focusing instead on peptide epitopes derived from M protein, or non-M protein-based antigens.

Peptide antigens derived from M protein are the most extensively studied vaccine candidates and all current GAS vaccine candidates in clinical trials are designed based on M protein-derived peptides [30]. The use of minimal epitope in peptide-based strategies provides the opportunity to select pathogen-specific protective sequences that do not stimulate cross-reactivity with human tissue [29]. Epitopes from the hypervariable N-terminus of M protein were shown to induce the highest level of bactericidal antibodies and did not cause autoimmune responses [30,32]. However, these epitopes are serotype-specific and only protect against specific GAS strains. Even with epitope combinations (multivalent M-type vaccine candidate), this approach is still hindered by N-terminus sequence diversity [51,52]. The use of highly conserved peptide epitopes from the C-repeat regions of M protein allow for the development of a broadly protective vaccine, but this strategy is limited by the poor immunogenicity and efficacy of these epitopes [31,53]. Therefore, effective delivery systems are needed to improve the efficacy of peptide-based GAS vaccines.

Potential non-M protein targets for vaccine development were successfully identified through advanced genome-wide analysis and bioinformatic (genomics, proteomic, and immunomic) tools and technology [30,54,55]. Numerous virulence factors that serve as potential vaccine candidates have been identified; for example GAS carbohydrate, surface-bound C5a peptidase (SCPA), fibronectin-binding protein streptokinase, serum opacity factor (SOF), streptococcal pyrogenic exotoxins (Spe), pilus, interleukine-8 (IL-8) protease (SpyCEP), streptococcal secreted esterase (Sse), G-related alpha2-microglobulin binding protein (GRAB), and the metal transporter of Streptococcus (MtsA) [54,56,57]. Combinations of several of these antigens in vaccine formulations have also been shown to boost bactericidal activity by inducing antibodies that target different GAS biological functions [30]. However, when a combination of streptolysin O, SpyCEP, SCPA, arginine deiminase (ADI) and trigger factor (TF), adjuvanted with alum, was compared to immunisations with full-length M protein, only the M protein provided protection following subcutaneous challenge in mice models of infection [58]. Thus, regardless of the discovery of non-M protein antigens, M protein is still a major virulence factor determinant for type-specific immunity and primary antigen.

## 3. Major Antigen Targets for GAS Vaccines

As the M protein is the major target in vaccine development, GAS antigens have been broadly classified as M protein, non-M protein, and carbohydrate-derived antigens.

### 3.1. GAS M Protein

**Figure 1:**
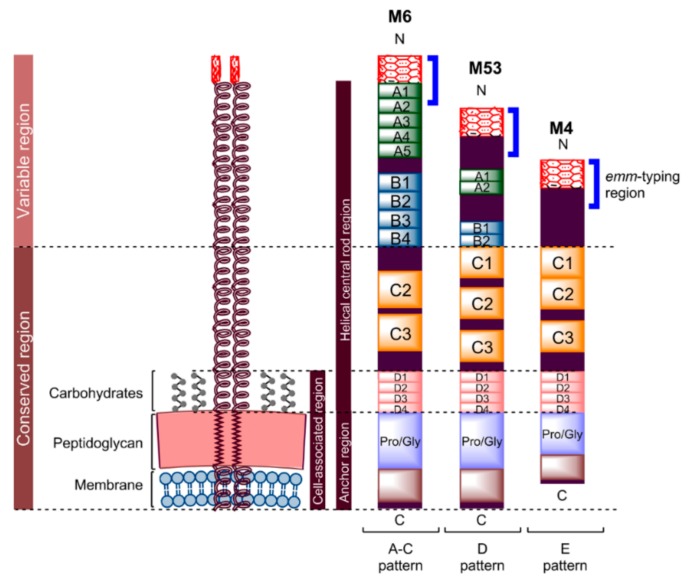
Schematic representation of M protein. All M proteins are similarly organised; they include a hypervariable N-terminus, variable central region, and highly conserved C and D repeats, though M protein varies in length depending on the pattern type [51]. M proteins may have a long A-C pattern (e.g., M6), intermediate D pattern (e.g., M53) or short E pattern (e.g., M4), with an average residue of 444, 355 and 316, respectively. The N-terminus non-helical region of M protein contains a negatively charged amino acid sequence (variable in length) adjoining the A-repeat region [11]. This region enables antibody recognition and electrostatic repulsion with phagocytes. A- and B-repeat regions have different sizes and numbers of repeat sequences between different M proteins [10,31,51]. On the other hand, the C-repeat region has a different number of repeat sequences, but with similar sequence identity. This conservation in sequence increases with the D-repeat region. These four regions form a helical central rod for M protein. The non-repeating block containing an excess of proline and glycine amino acids adjacent to the D-repeat region enables the M protein to traverse the cell wall. Whereas, 20 hydrophobic and six charged amino acids at the end of the C-terminus help the protein to extend along and anchor to the bacterial cytoplasmic membrane [61]. This cell-associated region is shorter in the E pattern of M protein compared to the other types, with 29 residues instead of 48 [51].

M protein (Figure 1) is a hairlike, coiled-coil homodimer surface-anchored protein encoded by the *emm* gene. It forms the basis for the serological differentiation of GAS strains [56,59,60]. M protein is the major virulence factor of GAS and it plays a vital role in preventing opsonophagocytosis in the host’s immune system [33,61] by binding complement regulatory proteins, serum proteins (fibrinogen and albumin) and immunoglobulins [11,51]. In addition, M protein also mediates epithelial adherence and invasion and assists GAS survival in the presence of neutrophils [1]. M protein serotypes are closely associated with categories of human infection [60,62]. For example, serotype M1 and M3 GAS strains often cause pharyngitis and invasive infections, while M28 GAS strains are responsible for puerperal sepsis and neonatal GAS infections. These serotypes are not equally distributed worldwide. For instance, M2, M4 and M12 strains are a common cause of invasive infections in the United States and other developed countries in contrast to the other parts of the world [63].

M protein consists of an N-terminus region, followed by distinct A-, B-, C-, and D-repeat regions. It is anchored to the bacterial cell membrane by a C-terminus region (Figure 1) [10,59]. The M protein’s non-helical, N-terminus-adjoining A-repeat region contains an excess of negatively charged amino acids that result in a net negative charge to the region, which enables electrostatic repulsion between GAS and phagocytes [11]. The helical central rod region formed by the four distinct segments assists the repulsion by acting as a shaft separating the negatively charged region from the bacterial surface. The A-repeat region at the N-terminus, which contains a highly variable amino-terminal, and the conjoining non-helical region define the GAS serotype (M type) and genotype (*emm* type) [55,64]. Although antibodies against this region can neutralise the negative charge and, consequently, enable the occurrence of phagocytosis, GAS counter-attacks this problem by performing antigenic variation on the N-terminus to avoid antibody recognition. More than 200 different GAS serotypes have been identified based on variation in the non-helical region and the A-repeat domain of the N-terminus [32]. The sequence variability becomes more conserved towards the C-terminus. However, antibodies/T-cells against some parts of the region downstream from the N-terminus may cross-react with human proteins and lead to autoimmune responses [10,65]. The risk of autoimmune response induced by streptococcal M protein (and its serotype diversity) is a huge obstacle in GAS vaccine development.

#### 3.1.1. Variable Region N-Terminal GAS Epitopes

As N-terminus epitopes are highly variable and geographically specific, vaccines that use them effectively are likely to employ a multivalent approach, with a variety of N-terminal antigens (from the most prevalent M types in the region or targeted population) combined by chemical synthesis or the recombinant approach [55,66]. Recombinant DNA technology is used to build multivalent recombinant genes that express multiple protective M protein-derived peptides (fusion proteins). Based on this strategy, multivalent vaccines have been developed and a few of them have reached clinical trials (Table 2) [67,68]. Despite these efforts, the variability in circulating *emm* type only provides a temporary reduction in GAS infection rates until new strains are introduced to the vaccinated regions. Extensive epidemiological investigations to identify emerging *emm* types and factors that affect their distribution are crucial in aiding the design of future multivalent vaccines with better coverage [69].

##### Advanced Preclinical and Clinical Trials

GAS vaccine candidates have been developed based on variable regions, and some of these are currently in human clinical trials (Table 2).

The 6-valent vaccine candidate developed by James B. Dale and colleagues contains the amino-terminal peptides identified from six epidemiologically important GAS serotypes: 1, 3, 5, 6, 19 and 24 [71]. These serotypes were responsible for over 30% of pharyngitis infection cases and about 56% of RF cases in the United States (1988–1990). Primary Phase 1 studies of the 6-valent GAS vaccine adjuvanted with aluminium hydroxide was performed in healthy volunteers to test the safety and preliminary efficiency of the vaccine [71]. This vaccine was effective in inducing opsonic antibodies against all six GAS serotypes, without triggering tissue cross-reactivity or clinical complications. However, it was argued that these findings were biased due to the small scale and open-label design (non-blinded experiment) of the Phase 1 trial. Thus, large-scale clinical trials are necessary to verify the complete safety of the 6-valent vaccine. Currently, no information is available on the continuation of this study.

Meanwhile, a new multivalent vaccine containing M protein-based epitopes covering a larger number of epidemiologically important GAS serotypes has been designed. The 26-valent vaccine comprised N-terminal epitopes from GAS serotypes, which were selected based on current epidemiology studies of GAS infections in the United States and Canada, including but not limited to pharyngitis, invasive infections and RF [76]. In addition, special serotypes (e.g., s M19 and M24), which were historically reported to induce RF, were also incorporated into the vaccine. The 26-valent vaccine was built based on four recombinant proteins, each including six or seven epitopes derived from the N-terminal region of M proteins. The preclinical 26-valent vaccine (adjuvanted with alum) study was conducted in rabbits. The results demonstrated the vaccine’s ability to induce type-specific antibodies against 25 out of the 26 GAS serotypes, without triggering human tissue cross-reactivity [67]. In the Phase 1 clinical trial, the formulated 26-valent vaccine (known as StreptAvax) was intramuscularly injected into 30 healthy adult volunteers to evaluate the safety and immunogenicity of the vaccine [72]. The vaccine was highly immunogenic and induced the production of bactericidal antibodies without demonstrating any adverse side-effects. Immunogenicity and the safety of this vaccine candidate was further confirmed in Phase 2 clinical trials [72,73].

Due to the efficacy of the 26-valent GAS vaccine demonstrated in preclinical and clinical trials, a novel 30-valent vaccine was constructed in 2011. The vaccine is comprised of N-terminal epitopes of M protein from 30 GAS serotypes, which were selected based on epidemiology studies of GAS infections in the United States and Europe [68,72,76,77,78]. Compared with the previous 26-valent vaccine, the 30-valent vaccine candidate covered broader and more epidemiologically -important GAS serotypes that cause uncomplicated pharyngitis, invasive complications and RF. The vaccine is comprised of four recombinant proteins, each containing seven or eight different M protein-derived epitopes. The 30-valent vaccine, adjuvanted with alum, was tested in a preclinical study to evaluate its safety and immunogenicity. The vaccine was highly-immunogenic and induced diverse opsonic antibody production against the 30 GAS serotypes and against several “non-vaccine” serotypes. Thus, the efficacy of the 30-valent vaccine exceeded expectations based on the design of type-specific M protein-based epitopes. A Phase 1 clinical trial of the 30-valent vaccine was planned for 2015 [79]; however, no further information is available regarding this study.

The intensive analysis of 176 *emm* GAS types [80] has produced a new *emm* cluster-specific system, which could provide protection to different *emm* types within the same cluster [75,81]. Five N-terminal M peptides from the E4 *emm* cluster, containing 17 GAS *emm* types that are prevalent in the United States, were selected [75]. This 5-valent E4 recombinant peptide vaccine was administered to rabbits to evaluate immunogenicity. The recombinant peptide induced antibody production that cross-reacted to all E4 M peptides and was opsonic to the 17 GAS E4 *emm* types. Cluster-specific systems have the potential to provide broader protection against the majority of clinically relevant GAS *emm* types, without the need to incorporate dozens of epitopes into vaccine antigens [81].

#### 3.1.2. Conserved Region M Protein Epitopes

Vaccine candidates that use small peptide epitopes derived from the extracellular, conserved C-repeat region of M protein have the potential to provide protection against a broad spectrum of GAS strains [11,55,82]. The C-repeat region is highly conserved between different GAS strains, and antibodies produced against it can protect against multiple GAS *emm* types. However, problems regarding the immunogenicity and cross-reactivity of this region with human heart tissue have been the main concerns for vaccine development [30,62]. Therefore, it was critical to identify minimal protective antigen sequences from the C-repeat region that are non-auto-immunogenic.

Epitope p145 (LRRDLDASREAKKQVEKALE), a 20-mer peptide from the C-repeat region of the M protein, was identified as recognisable by the human sera antibodies of most adults living in GAS endemic areas [49,62,83]. Furthermore, mice immunised with p145 were able to produce opsonic antibodies against GAS [49,83]. However, a T-cell epitope found within the p145 sequence was computationally predicted to stimulate cross-reactivity with human heart and keratin tissue, implying the risk of inducing autoimmune disease [62,84]. Hence, the 20 amino acid sequence of p145 was split into shorter peptides in search of minimum B-cell epitope that was capable of stimulating humoral immunity [49,53]. The identified J8i epitope (SREAKKQVEKAL) was flanked by two sequences from GCN4 DNA binding protein to produce J8 epitope (QAEDKVKQ**SREAKKQVEKAL**KQLEDKVQ), which was able to maintain its native helical conformation [31,49]. J14 epitope (KQAEDKVK**ASREAKKQVEKALE**QLEDKVK), a close analogue of J8, was also designed in a similar manner [49].

Subcutaneous vaccination with either J8 or J14 peptide epitope triggered the production of serum opsonic IgG antibodies in mice, which provided protection against systemic challenge. This also demonstrated cross-protective activity against broad types of GAS M proteins [49,55]. Furthermore, neither J8 nor J14 are homologous to known human proteins and they did not cross-react with human tissues [49,85,86], nullifying the risk autoimmune response and suggesting that these epitopes would be safe for human use. Therefore, J8 and J14 epitopes have been used in many studies on GAS vaccine development [29,87,88,89,90,91].

#### Clinical Trial

In 2003, Michael F. Good and colleagues designed a vaccine, J8-DT, containing the minimal B-cell epitope J8 and diphtheria toxoid (DT), with human-compatible adjuvants (SBAS2 or alum) [92]. Upon subcutaneous injection, J8-DT was able to stimulate the production of opsonic antibodies, which protected mice against intraperitoneal disease challenge. DT, as a carrier protein, induced T-helper cell response. B-cell responses induced by J8-DT were long-lasting and resulted in the generation of specific memory B-cells (MBC) and long-lived plasma cells [93]. This vaccine was further evaluated to confirm its immunogenicity and safety. The double-blinded Phase I pilot study of J8-DT (also known as MJ8VAX) was successfully completed in 2018: antibody response against J8 was produced in humans and no complications or side-effects were reported [94].

#### 3.1.3. Combined Epitopes

A vaccine that incorporates a combination of peptides from the C- and N-terminal region of M protein may provide better protective immunity and broader coverage due to the combined functions of serotypic and conserved epitopes [62]. Each epitope could be designed to elicit protective antibodies, T-cell involvement, or both, without stimulating cross-reactivity to host proteins [85,95]. Two such vaccines were designed: the first was produced by the modification of peptide epitopes with the acryloyl group and then polymerisation [85]; and the second, as a large polyepitope recombinant protein [96]. Unfortunately, the use of complete Freund’s adjuvant (CFA) was required for efficacy. CFA is a gold-standard adjuvant; however, it is too toxic for human use. Therefore, further modification of these systems would be required to improve safety and efficacy.

### 3.2. Non-M Proteins

Apart from M protein, numerous GAS proteins have been identified and investigated as new antigens for vaccine development (Table S1). In the last few decades, various strategies have been applied to identify protective non-M protein GAS antigens [59,97,98,99,100]. Interestingly, as most of the non-M protein antigens can downregulate anti-GAS immune responses, the vaccine candidates usually do not bear the whole proteins, but rather employ peptides derived from them [101]. The peptides are also chosen to (a) eliminate unnecessary antigenic material, which does not contribute to a protective immune response, and may induce deleterious immune responses, and because (b) minimal epitope-based vaccine is expected to prevent acute infections while significantly minimizing any potential risk of post-streptococcal autoimmune sequelae [101,102,103].

#### 3.2.1. Fibronectin-Binding Proteins

Fibronectin-binding proteins are involved in bacterial attachment to host cells and serve as potential vaccine targets against GAS infections [104] because neutralising antibodies against these adhesive proteins should prevent bacterial attachment and inhibit colonisation. Fibronectin-binding protein F1 (Sfb1) has been identified as a highly conserved surface protein of GAS, expressed by 73% of clinical isolates belonging to different serotypes and strains that do not induce cross-reactivity with human tissues [105,106]. Sfb1 is able to mediate bacterial attachment to host cells and the internalisation of GAS into non-phagocytic cells [104,107,108]. It is also capable of interfering with host macrophage activation by binding to the Fc fragment of host immunoglobulins and avoiding the host’s clearance mechanisms [109]. Mice intranasally immunised with either Sfb1 alone, or Sfb1 adjuvanted with cholera toxin B (CTB) produced systemic IgG and lung mucosal IgA responses, which provided protection against a lethal intranasal GAS challenge [105]. However, further studies showed that Sfb1/CTB intranasal vaccination was unable to elicit either opsonising antibodies or systemic immunity against bacterial colonisation and invasion to internal organs after subcutaneous GAS challenge [104]. Other highly conserved fibronectin-binding proteins, such as FBP54 or FbaA, also induced strong immune responses in mice, with FbaA having a similar ability to GAS M protein in generating immune responses and immunoprotection [110,111].

Minimal fibronectin-binding repeats (FNBR) with 148 amino acids within the binding domain of Sfb1 were identified [106]. A dual-antigen component GAS vaccine consisting of Sfb1 FNBR and M protein J8 epitope inside a lipid core peptide (LCP) delivery system was designed to stimulate both mucosal and systemic immunity against bacterial colonisation and invasion, and to induce opsonising antibodies for bacterial clearance [64]. The LCP-J8-FNBR vaccine (adjuvanted with macrophage-activating lipopeptide (MALP-2), a TLR2/6 agonist) induced strong systemic and mucosal immune responses upon intranasal immunisation in mice and provided complete protection following a lethal pulmonary challenge. When antigenic linear B-cell (FNBR-B) and T-cell (FNBR-BT) epitopes were identified within the FNBR of Sfb1 [106], a bivalent vaccine containing FNBR-B and J14 epitope incorporated into an LCP delivery system (LCP-J14-FNBR-B) [89] was formulated with a mucosal adjuvant BPPCysMPEG (a derivative of MALP-2). LCP-J14-FNBR-B stimulated immune responses at a low dose (similar to those observed in LCP-J8-FNBR) [112].

#### 3.2.2. Interleukine-8 (IL-8) Protease (SpyCEP)

Interleukin 8 (IL-8) cleaving enzyme SpyCEP is a highly conserved GAS cell wall-anchored protein [97,113]. SpyCEP purified from an M81 GAS strain cleaved the C-terminus of IL-8 at residues Glu59 and Arg60 and inactivated its chemotactic properties [114]. Thus, the upregulated expression of SpyCEP can hinder neutrophil-controlled killing and strengthen the migration of bacteria from the surface of host skin to deep tissue.

Sriskandan and co-workers proved that anti-SpyCEP antibodies can protect IL-8 from degradation by the enzyme [115]. Therefore, epitopes derived from SpyCEP have been considered as potential vaccine candidates against highly virulent GAS strains. Recently, Pandey et. al. identified a variety of SpyCEP epitopes (S1–S6), which upon conjugation to DT, were able to protect IL-8 from cleavage [102]. Among the minimal SpyCEP epitopes tested, S2 conjugated to DT demonstrated the best ability to induce anti-SpyCEP antibodies, allowing neutrophil accumulation at infection sites. When this epitope was combined with J8/DT and formulated in alum, the vaccine (J8-DT/S2-DT) provided complete clearance of systemic infection in mice model and was more efficient than J8-DT.

#### 3.2.3. Surface-Bound C5a Peptidase (SCPA)

Surface-bound C5a peptidase (SCPA) is a large surface protein expressed by most GAS serotypes [116]. SCPA helps in cleaving complement-derived C5a chemokines (the signalling proteins responsible for the initiation of early inflammatory events) and removing the leukocyte-binding site C5a [116,117]. When SCPA inactivates chemokines, the infiltration of phagocytes is delayed and the clearance of bacteria from mucosal and sub-dermal surfaces is hindered. This can result in the establishment of infection in the host [117]. Intranasal immunisation of mice with the recombinant subunit SCPA49 mutated protein (derived from serotype M49) strain induced the production of antigen-specific salivary secretory IgA and serum IgG [116]. In addition, mice immunised with mutated SCPA protein produced antibodies that were able to clear GAS from the oral-nasal mucosa and lung [118,119].

#### 3.2.4. Other Potential Antigens

Protein G-related α2-macroglobulin binding protein (GRAB) is one of the conserved GAS virulence factors that is responsible for the inhibition of host proteases and immune responses [120]. On the other hand, the metal transporter of Streptococcus (MtsA) is a lipoprotein, which is a part of an ATP-binding cassette (ABC) transporter complex that works as a metal binding protein [121]. EIN19 and EKL24 are peptide epitopes derived from MtsA and GRAB, respectively [54]. These peptides were conjugated to keyhole lymphocyanin (KLH) carrier protein, but did not induce effective immune responses.

Superoxide dismutase (SOD) enzyme was identified as a potential antigen because it is highly conserved in GAS [57]. GAS SOD is responsible for detoxifying and protecting GAS against reactive oxygen species (ROS). The gene that encodes for SOD is *SodA*. Inactivation of this gene was able to limit the growth capacity of GAS, especially in aerobic conditions, due to its high sensitivity to oxidative stress [57,122]. The antibodies elicited against the *SodA* gene are present in high levels in patients from GAS-endemic areas. Mice subcutaneously vaccinated with recombinant *SodA* produced high antibody titres and were able to elicit a moderate opsonic response against GAS [123]. However, immunisation did not protect against intraperitoneal challenge with GAS. Thus, it was suggested that in contrast to M protein antibodies, the antibody response to SodA during natural infection may not offer protection. However, it is important to note that this assumption was made based on the outcome of rodent studies.

### 3.3. GAS Carbohydrate (GAC)

Aside from M protein gene (*emm*) typing, another method for GAS serotype classification is based on the expression of unique, cell wall-anchored carbohydrate antigens [124,125]. Although the GAC antigen occupies approximately half the weight of the GAS cell wall [126], the biological function of GAC antigen is still not clear [127]. This carbohydrate is expressed by all GAS serotypes [127] and has been considered as a potential antigen for a universal vaccine against GAS infections. GAC is made up of a poly-rhamnose backbone with an immunodominant *N*-acetylglucosamine (GlcNAc) side chain. The side chain from GAC has been identified as a target in all rapid diagnostic assays for GAS infections [127]; however, it has the potential to induce autoimmune responses in humans [128,129,130]. Controversially, it was demonstrated that children are able to produce opsonic anti-GAC antibodies without autoimmune responses being triggered [131]. This observation prompted Zabriskie and colleagues to develop a conjugate vaccine consisting of GAC and tetanus toxoid (TT) (GAC-TT) [132]. The GAC-TT conjugate was able to stimulate a high level of opsonic anti-GAC antibodies that do not cross-react with human tissues or skeletal proteins. However, a recent study established that the GlcNAc side chain is not a universal GAS virulence factor, which may explain the variability of reported GAC cross-reactivity [133].

Kabanova et al. evaluated the immunogenicity of minimal GAC-derived antigenic determinants [134]. Four pure and homogeneous oligosaccharides (hexa- and dodeca-saccharides) with well-defined structures were designed, chemically synthesised and conjugated to CRM197 (an enzymatically inactive non-toxic form of DT). The conjugate triggered immune responses protecting mice against GAS challenge with the same efficacy as the native conjugates containing natural GAC. This has provided a substantial starting point for the potential future development of safe carbohydrate-based GAS vaccines.

## 4. Development of Adjuvants and Delivery Systems for GAS Peptide

Short peptides are not effectively recognised by DCs and macrophages, and therefore, require additional immunostimulant or/and delivery systems to enhance their immunogenicity and efficacy. Delivery systems protect peptides from enzymatic degradation and improve vaccine uptake by APCs without using specific receptor recognition. The efficacy of adjuvant/delivery system depends on its size, shape, surface characteristics, and morphological and physiochemical properties [25]. Adjuvants often promote vaccine uptake through toll-like receptor (TLR) recognition on APCs or via the activation of other immunostimulatory pathways. The development of adjuvants is hindered by their toxicity, hypersensitivity reactions and the duration of their effectiveness. Although there are a large number of experimental adjuvants available, most of them are not suitable for human use regardless of their excellent immunostimulatory properties because of the serious adverse effects they can cause [25]. This includes CFA and CTB, which are gold-standard adjuvants used in animal models. On the other hand, the limited supply of commercial adjuvants (aluminium salt, liposome-based adjuvants (virosome and AS01), recombinant CTB, emulsions (MF59, AS03, and AF03), RC-529 synthetic monophosphoryl lipid A, and Montanide ISA-51) are approved only for specific vaccines and within certain countries [25,27]. Therefore, the discovery of safe, effective adjuvants and delivery systems is crucial in developing the protective responses of vaccines against weak antigenic molecules, such as peptides.

### 4.1. Peptide Lipidation

**Figure 2:**
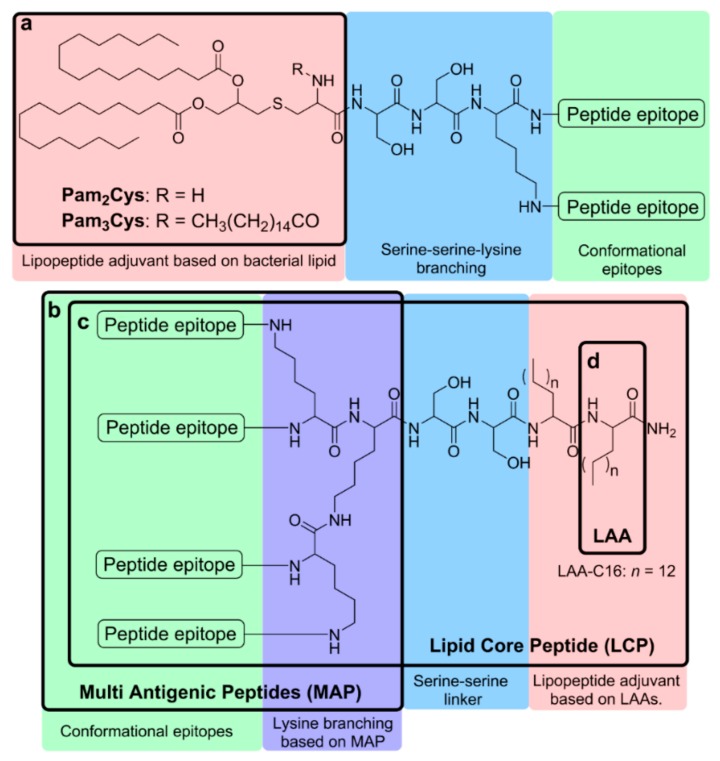
Schematic representation of lipopeptide-based vaccines bearing (**a**) Pam_3_Cys and Pam_2_Cys, (**b**) a lipid core peptide (LCP) system, (**c**) a multiple antigen peptide (MAP) system, and (**d**) lipoamino acids LAAs.

Peptide lipidation is an approach to produce self-adjuvanting synthetic lipopeptides by conjugating lipids to peptide epitopes [27]. This strategy mimics lipoproteins, which are common components of bacterial cell walls and strong immunogens [135]. In addition, the amphipathic character of lipopeptides enables their self-assembly into nanosized particles [87]. It has been confirmed that synthetic lipopeptides produced by chemical conjugation induce a high degree of immunogenicity with few or no adverse side-effects [136,137,138]. Lipidation enhances peptide hydrophobicity, the peptides’ ability to permeate biological membranes via passive diffusion and stability against enzymatic degradation [26,27]. Moreover, lipidic moieties are often recognised by TLRs, which instigates effective uptake of peptide antigen by APCs and triggers APCs maturation [139,140,141]. Thus, lipidation has been widely used for the delivery of peptide-based GAS vaccines (Figure 2).

#### 4.1.1. Pam_3_Cys and Pam_2_Cys

Tripalmitoyl-S-glyceryl cysteine (Pam_3_Cys) and dipalmitoyl-S-glyceryl cysteine (Pam_2_Cys) are lipid moieties with self-adjuvanting properties [142,143]. Lipopeptides containing either Pam_3_Cys or Pam_2_Cys are able to induce humoral and cellular immune responses against conjugated peptide epitopes without the presence of additional adjuvant through the activation of TLR2/1 [144]. Pam_3_Cys is a synthetic analogue of the N-terminal moiety of lipoproteins of Gram-negative bacteria. Pam_3_Cys was approved by the FDA for the self-adjuvanting *Neisseria meningitidis* vaccine [143]. However, Pam_3_Cys-conjugated lipopeptides suffer from poor water solubility and usually require the addition of a water-solubilising moiety [142,143]. Pam_2_Cys is a close analogue of Pam_3_Cys, but lacks one palmitoyl group. It is derived from the macrophage activating lipopeptide-2 (MALP-2) of *Mycoplasma fermentans*. Pam_2_Cys has improved water solubility and maintains the ability to stimulate humoral and cellular immune responses [140]. In contrast to fully lipidated Pam_3_Cys, Pam_2_Cys bears one free amine group that, according to a recent study, is able to interact with the polar residues of the TLR2 hydrophobic lipid-binding pocket, which improves its uptake by APCs [145].

Subcutaneous immunisation with Pam_3_Cys and Pam_2_Cys conjugated to GAS variable and conserved epitopes derived from M protein induced high antigen-specific IgG antibody responses in mice. The produced antibodies were able to bind strongly to the cell surfaces of a variety of highly virulent GAS serotypes [146]. Pam_2_Cys conjugated to J14 and P25 (a universal helper CD4+ T-cell epitope; KLIPNASLIENCTKAEL) induced J14-specific IgA antibody responses and protect mice from respiratory GAS challenge upon intranasal immunisation [147]. A recent study demonstrated that mixed micelles formulated with J8-dipalmitoylglutamic acid and Pam_2_Cys enhanced the delivery of antigens and adjuvant to the lymph nodes, significantly improving systemic and mucosal antibody responses in mice [148].

A simplified analogue of Pam_2_Cys was designed by replacing the Cys and glycerol moieties in Pam_2_Cys with Ser residue [139,149]. Dipalmitoyl serine (DPS) demonstrated an adjuvanting ability similar to Pam_2_Cys and an affinity to TLR2. Similarly, an analogue of Pam_3_Cys was designed by the chemical binding of three dodecanoylated lysine moieties, which upon conjugation with J8 was able to induce the same level of antibody titres as DPS [149].

#### 4.1.2. Lipoamino Acid (LAA) and Lipid Core Peptide (LCP)

Lipoamino acids (LAAs) are alpha-amino acids with long alkyl side chains. They possess structural similarities to lipids combined with amino acids. LAA-based lipopeptides induce immune responses through TLR2 and DCs activation [84,136]. LAAs are very versatile molecules and can be conjugated to peptides at N-amino or C-carboxy terminals and incorporated anywhere in the sequence. Thus, the length, number, branching and type of the lipidic chains in a peptide can be modified, which consequently affects the lipophilicity, solubility and stability of the conjugate [83,142]. To induce strong immune responses, at least two copies of LAAs have to be conjugated to an antigen. The lipopeptides formed have an amphiphilic structure and can self-assemble into particles. The size of the nanoparticle may be controlled by the number of LAA residues and the length of the LAA alkyl chain [29,150,151]. Moreover, the induction of immunological responses can be improved by changing the structural arrangement of epitopes and lipid moieties in a single construct [136,152].

Multiple antigen peptide (MAP) systems enable the incorporation of multiple copies of peptide epitopes in a single construct, which stimulates the generation of a higher antibody response [153]. MAP is an amino acid-based dendrimer with a poly-lysine core. Each lysine has functional side-chains (α- and ε-amino groups) available for conjugation to peptide antigens. The lysine carrier in MAP allows for the conjugation of multiple copies of the same or different peptide epitopes. By using MAP, the antigen can be protected against degradation, in addition to improving antigen presentation and recognition by APCs [26]. Immunogenicity induced by MAP is significantly higher than with monomeric peptides [26,27,29,154], possibly due to the clustering effect of B-cell receptors [155]. MAP also enables the incorporation of both B-cell and T-helper epitopes in the same construct, which together can be taken up by APCs to target humoral responses [27]. The addition of T-helper epitopes with peptide antigen enhances antigen-specific immune responses, and more importantly, memory responses. Activated T-helper cells encourage the stimulation of B-cell proliferation and induce antibody production. MAP has been applied in GAS vaccine delivery as a part of lipid core peptide (LCP) systems.

LCP systems incorporate non-microbial LAA-based lipopeptide adjuvants, the branching moiety of MAP and peptide antigens into a single molecular entity as a vaccine delivery system [89,90,156]. Each component in this system can be easily modified: the number of LAAs, as well as the length of their alkyl chains, can be altered, one or more lysines can be incorporated to regulate the branching level, and multiple copies of a single, or variety of epitopes can be incorporated [29]. LCP vaccine candidates are more stable at room temperature and against peptidase degradation than related peptides or proteins. The incorporation of J8 epitope in LCP constructs induced opsonic antibody production upon immunisation and provided protection in mice against GAS challenge without any additional adjuvant [157]. The incorporation of multiple GAS epitopes in the LCP system was also evaluated. Mice intranasally immunised with the vaccine candidates induced highly opsonic antibody production against incorporated epitopes [53]. Toth and colleagues investigated a library of vaccine candidates against GAS incorporating J14, P25 and LAAs [152,158,159]. The number and length of LAAs, the spacing between lipid chains and the structural arrangement of the components were all altered to optimise the LAA-based lipopeptides. Following intranasal immunisation in mice, the induction of J14-specific IgG antibody production varied between vaccine candidates based on the point of lipid moiety attachment and the orientation of the J14 and P25 epitopes. The length of LAA alkyl side chains also had an effect on antibody titres. The conjugate arrangement with two copies of 2-amino-d, l-hexadecanoic acid (C16) at the C-terminal, N-terminus P25, and J14 on the lysine side chain was the most effective. Antibodies produced after immunisation with this vaccine candidate (J14-LCP) were able to inhibit bacterial growth and reduce throat colonisation after respiratory GAS challenge. When a similar strategy was used to incorporate J14 and 8830 N-terminus GAS epitope into LCP, the conjugate, which formed the smallest nanoparticles (~10 nm), induced greater antigen uptake by APCs, enhanced their maturation and triggered significantly higher antibody titres than analogues forming bigger nanoparticles (100 nm) [160]. The produced antibodies were able to bind to endemic GAS strains. LCP peptide vaccine can also be incorporated with other adjuvants or delivery systems (e.g., *N*-trimethyl chitosan (TMC) [161] and liposome [162]) to improve immunogenicity.

### 4.2. Glycolipid

Carbohydrates can be used as carriers for peptide epitopes in a manner similar to lysine dendrimer. Several peptides can be conjugated to carbohydrate hydroxyl groups using a variety of linkers [163,164,165]. Carbohydrates help to reduce enzymatic degradation of attached peptides, leading to potentially improved immune responses against incorporated peptide antigens [166]. In contrast, lipidated carbohydrates can act as adjuvants (e.g., Lipid A). The combination of peptide antigens with lipids and carbohydrates (liposaccharides) was examined for the development of synthetic GAS vaccines [167]. Palmitoylated monosaccharides and disaccharides conjugated with J8 were able to induce antibody production in mice in-line with CFA-adjuvanted J8 [168]. In another design, monosaccharides bearing multiple copies of J8 or J14 replaced polylysine branching in the LCP system [165]. These vaccine candidates were able to stimulate high antibody production, similar to CFA-adjuvanted epitopes.

### 4.3. Polymers

Polymers are widely used as immunostimulants and nanoparticulate vaccine delivery systems [169]. Numerous natural and synthetic polymers have been used to encapsulate antigens and adjuvants. Some of them, for example poly(lactic-co-glycolic acid) (PLGA), have been approved for clinical applications [170]. However, PLGA-based nanoparticles often suffer from slow cargo release, potential ‘burst release’ [171,172] and residual organic solvents following particle formation may denature antigens [173]. Additional cationic polymers are often required to achieve efficient vaccine delivery with PLGA. Chitosan is a biocompatible, biodegradable and mucoadhesive linear polysaccharide derived from chitin. It is comprised of N-acetylglucosamine and glucosamine units [174]. Chitosan-based nanoparticles can easily migrate to lymph nodes and trigger a strong humoral immune response [175]. TMC-based nanoparticles have been shown to be a promising delivery system for mucosal immunisation owing to the intrinsic adjuvant properties of TMC [174]. Indeed, TMC-coated PLGA nanoparticles bearing J14-LCP vaccine induced significantly higher humoral immune responses than uncoated PLGA/J14-LCP [176]. Interestingly, the ability of PLGA/J14-LCP nanoparticles to induce immuno-responses was also dependant on antigen localisation; LCP encapsulated in PLGA induced higher antibody titres than LCP-coated PLGA nanoparticles [177]. The highly cationic nanoparticles (300 nm, +40 mV) formed by LCP, dextran and TMC were the most efficient among the above mentioned polymeric systems in triggering opsonic antibody production against GAS following intranasal administration in mice [176]. TMC has also been applied in delivery systems comprising J8 epitope and a universal T-helper epitope, PADRE, which was first conjugated with anionic polyglutamic acid to introduce permanent anionic charge on an antigen, and then for its assembly into nanoparticles by mixing with cationic TMC [178]. These nanoparticles induced antibody production that was able to reduce bacterial burden in mice upon challenge with GAS M1 strain. The first polymer-peptide antigen conjugation approach was reported in 2010, where J14 epitope was conjugated to dendritic polyacrylate polymer. The conjugate was self-assembled into nanoparticles, which were as effective as CFA/J14 in inducing antibody production [179] through intranasal administration [180] in a size dependent manner following single dose administration [181], and the produced antibodies were opsonic against all tested GAS clinical isolates [182].

### 4.4. Liposomes

Liposomes were first discovered over half a century ago [183]. During the past 40 years, liposomes have attracted much attention as potential pharmaceutical carriers for the delivery of genes and drugs because they are biocompatible, biodegradable and are able to increase the potency and reduce the toxicity of drugs [184]. Liposomes as a drug delivery system can encapsulate hydrophilic and hydrophobic drugs owing to their unique structure: an aqueous core enclosed by phospholipid bilayer membranes. The advantages of liposomes as drug delivery systems include: (1) a variety of available lipid compositions exist that can be used for the optimisation of drug pharmacokinetics; (2) the ability to protect active ingredients from enzymatic degradation; (3) improvement to the therapeutic index of drugs; and (4) the possibility for drugs to target specific sites or immune cells [185]. In 1974, Allison and Gregoriadis first reported the capacity of liposomes to provoke immune responses to incorporated antigens [186,187]. Since then, many efforts have been made to develop liposomes as a vaccine delivery system, not only as antigen delivery carriers but also as a tool to improve the immunogenicity of peptide- and protein-based antigens. A variety of factors can influence immune responses caused by liposome-based nano-vaccines, including the composition, charge and size of liposomes, the presence of moieties targeting specific immune cells and the route of vaccine administration [188,189,190]. Generally, small liposomes (<200 nm) move to the lymph nodes through passive diffusion, where they are endocytosed by DCs whereas large liposomes (>500 nm) are engulfed by macrophages [188,191]. Thus, the size of liposomes determines the initial type of immune response.

Ghaffar et al. developed cationic liposomes as an intranasal delivery system for J14-LCP vaccine by anchoring the lipopeptide vaccine to the liposome membrane [162]. These liposomes induced higher systemic and mucosal antibody responses compared to antigen administered with standard mucosal adjuvant CTB. The influence of liposomal size was analysed, and the smallest unilamellar liposomes (70 nm) were more efficient in inducing humoral immune response than 140 and 400 nm liposomes [192]. Interestingly, large, multilamellar liposomes (150–1000 nm) were also very potent. When similar J14-LCP-anchored liposomes were coated with alginate and TMC, they were able to induce long-lasting antibody responses following oral administration. This was in contrast to uncoated liposomes and CTB-adjuvanted J14-LCP, which were not effective [161]. Liposomal vaccine carrying palmoylated J8 was also combined with a carrier protein. The carrier protein DT was not conjugated to antigen, but encapsulated into liposomes, to which palmoylated J8 was anchored [193]. The immune responses generated by this system were effective in reducing bacterial infection when immunised mice were challenged with GAS.

## 5. Conclusions

The prevalence of diseases, such as RF and RHD caused by GAS infections, has a huge impact on society and result in a high demand for GAS vaccines. This is especially true in developing countries, where GAS is most problematic. The development of GAS vaccines has historically focused on the M protein, which is considered to be major virulence factor in GAS infection. N-terminus fragments of the M protein, which have the ability to induce highly opsonic and bactericidal antibodies have been introduced into recombinant multivalent vaccines. One of these, the 26-valent vaccine, is currently in clinical trials and is showing great potential as a safe and highly immunogenic GAS vaccine, especially for the most common GAS infections diagnosed in the United States. However, this vaccine may not be as effective in other parts of the world due to the vast differences in GAS epidemiology. Therefore, vaccines based on the conserved region of M protein serve as more promising vaccine candidates in a global context. J8-DT’s success in the initial clinical trials places us one step closer to achieving protection against all GAS strains. Furthermore, other non-M protein antigens have also been investigated as potential vaccines; however, none of these candidates have entered clinical trials yet. They also may not provide as good protection as the M protein-based peptide vaccines.

Aside from the diversity of GAS serotypes and the cross-reactivity of GAS with human proteins, the lack of native animal model to study GAS pathogenesis is also a factor that has hindered GAS vaccine development. GAS is a human-specific pathogen, and rodent models are unable to establish significant oropharyngeal colonization of GAS or develop evidence of symptomatic infection. On the other hand, non-human primates have similar development and components of immune responses against GAS as humans; however, their use is typically limited to non-invasive GAS colonization, in addition to very limited access to the monkey model for research purposes and its high cost.

Not only is the choice of antigen crucial for successful commercial vaccine development, but so is the selection of the delivery system/adjuvant. While finding an effective and safe method of vaccine delivery has been challenging, the application of LCP, liposomes, carrier proteins or polymers can compensate for the limited immunogenicity of peptide epitopes and certainly improves vaccine efficacy by evoking systemic and mucosal humoral immune responses. Although reported delivery systems have the potential to be utilised to produce safe and effective self-adjuvanting vaccines against GAS infection, further efforts are required to test them in more advanced preclinical settings using non-human or human primates prior to human clinical trials.

In conclusion, it is feasible that research efforts to-date have paved the way for the construction of commercially available GAS vaccines. However, the timeframe for achieving this grand challenge will depend on how much pressure the public places on developing such a vaccine.

## Figures and Tables

**Table 1:** Clinical manifestations of Group A Streptococcus infection [1,2,3,9,10,11].

**Non-Invasive Diseases**		
**Diseases**	**Description**	**Symptoms**
Pharyngitis	Benign local throat infection. Common manifestation of GAS infection. Arises from complex host-pathogen interaction.	Sore throat, high fever, cervical lymphadenopathy, tonsil exudates, raised peripheral white cell count.
Tonsillitis	Benign local tonsil infection.	White/yellow spots on tonsils, sore throat, swollen jaw lymph glands, fever, bad breath.
Impetigo/Pyoderma	Benign local skin infection. Common in childhood. Arises from complex host-pathogen interaction.	Superficial, non-follicular, crusted lesion on the face and other exposed body parts.
Scarlet fever	Disease that can follow an episode of GAS-mediated pharyngitis. Commonly occurs in children.	Diffuse blanching rash on chest to abdomen, sandpaper-like texture to the skin.
Otitis media	Infection in the middle ear.	Otalgia, otorrhea, headache, fever, appetite loss, vomiting, diarrhoea, hearing loss, tinnitus, vertigo.
**Invasive Diseases**		
**Diseases**	**Description**	**Symptoms**
Cellulitis	Painful skin infection on deeper subcutaneous tissue. Frequent manifestation of invasive GAS. Incidence increases with age.	Fever, systemic toxicity, inflammation of the skin.
Pneumonia	Infection of the lung.	Difficulty in breathing, fever, appetite loss, abdominal pain, headache, chest pain, cough, cyanosis.
Necrotising fasciitis (flesh eating bacteria)	Rapidly progressing skin infection that causes a destruction of subcutaneous fat, tissue and muscle.	Fever, spared overlying skin, severe pain, violaceous, bullae and slough skin, shock, multi-organ failure.
Streptococcal toxic shock syndrome (STSS)	Associated with GAS necrotising fasciitis.	High fever, hypotension, rash, coagulopathy, respiratory distress syndrome, renal failure, hepatic impairment, multi-organ failure.
Erysipelas	Painful skin infection. Frequent manifestation of invasive GAS. Incidence increases with age.	Fever, systemic toxicity, clear demarcated red inflammation, formation of superficial bullae.
Meningitis	Inflammation of the meninges. GAS is an uncommon cause of meningitis.	Fever, headache, stiff/sore neck, vomiting, appetite loss, tiredness, drowsiness, irritability.
Bacteraemia/septicaemia	Blood poisoning due to the presence of bacteria/toxin in the blood.	Sudden high fever with chills, nausea, vomiting, diarrhoea, abdominal pain, confusion, anxiety, tachycardia.
Lymphangitis	Infection of draining lymphatic tracts.	Tender linear streak extending from the site of infection.
Septic arthritis	Painful infection of the joint following episode of GAS-mediated pharyngitis.	Fever, enlarged joints.
Puerperal sepsis	Infection resulting from the birthing process. Frequent cause of death in the pre-antibiotic era.	Postpartum endometritis, peritonitis, septic, thrombophlebitis/bacteraemia without focus, fever for 24 h or recurring after childbirth/abortion.
**Post-Infectious Diseases**		
**Diseases**	**Description**	**Symptoms**
Rheumatic fever (RF)	Inflammatory disease caused by cross-reactive antibodies induced after GAS infection.	The combination of fever, polyarthritis/arthralgia, carditis, erythema marginatum, chorea, subcutaneous nodules, and mitral/aortic valve damage. Can turn into RHD.
Rheumatic heart disease (RHD)	Inflammation caused by cross-reactive antibodies induced after GAS infection leading to permanent damage to heart tissue and valves.	Tissue inflammation that results in carditis, valvulitis, arthritis, chorea, erythema marginatum and/or subcutaneous nodules.
Post-streptococcal glomerulonephritis (PSGN)	Inflammation of the glomeruli in the kidney caused by a build-up of immune complexes induced by GAS infection. Follows an episode of GAS-mediated pharyngitis/pyoderma.	Rapid onset of gross/microscopic haematuria oedema, hypertension, and encephalopathy.
Post-streptococcal reactive arthritis	Syndrome of polyarthritis that differs from acute RF/carditis.	Range of smaller joints unreactive to anti-inflammatory treatment.
Paediatric autoimmune neuropsychiatric disorder associated with GAS infection (PANDAS)	Cross-reactive antibodies induced after GAS infection that interferes with basal ganglia function causing symptoms of exacerbation in children. The existence of this disease is controversial.	Children with tie/obsessive compulsive disorder (OCD) worsened/developed after GAS infection.

**Table 2:** Multivalent vaccines derived from the N-terminus of GAS M proteins in advanced preclinical and clinical trials.

Name	Stage of Development			Comments	Ref.
	Preclinical	Phase 1	Phase 2		
6-valent vaccine	9 white rabbits IM injection	28 healthy adults	NI	Tolerable. No human tissue cross-reactivity. No clinical complications. Limited by small-scale trials.	[70,71]
26-valent vaccine	3 white rabbits IM injection	30 healthy adults IM injection	90 healthy adults IM injection	No evidence of RF or human tissue cross-reactivity. Highly immunogenic No control group was set.	[67,72,73]
30-valent vaccine	12 white rabbits IM injection	NI	NI	Highly immunogenic. Potential efficacy against non-vaccine-targeted GAS serotypes	[68,74]
5-valent E4 vaccine	3 white rabbits IM injection	NI	NI	Highly immunogenic. Potential to provide broad protection against GAS	[75]

NI: No further information is available; IM: intramuscular.

## Supplementary Materials

The following are available online at https://www.mdpi.com/2076-393X/7/3/58/s1, Table S1. Non-M protein vaccine candidates.
