# Supplementary material for: Recent Advances in the Development of Peptide Vaccines and Their Delivery Systems against Group A Streptococcus
Source: Vaccines (Basel). 2019 Jul 1;7(3):58. doi: 10.3390/vaccines7030058 (PMC6789462; doi:10.3390/vaccines7030058)
Supplement: Supplementary file 1 [file vaccines-07-00058-s001.pdf]

Table S1. Non-M protein vaccine candidates.

Non-M protein-based Vaccine Candidate	Vaccine candidates	Antigens	Adjuvants/ Delivery System	Stage of Development	Comments	Ref.
GAS Carbohydrate (GAC)	GAC-TT	GAC isolated from strain D58X, an M protein–negative mutant	Alum	Animal study: mice, NAS, SC	Immunized mice were protected against systemic or nasal challenge with GAS and no cross-reactivity was observed.	[1]
	Oligosaccharide	Synthetic oligosaccharides	Alum	Animal study: mice, IP; rabbit, SC	The synthetic conjugate vaccine candidates have similar efficiency conjugates of natural GAC.	[2]
Surface-bound C5a Peptidase (SCPA)	SCPA49	Recombinant SCPA49 mutated protein	-	Animal study: mice, NAS	SCPA49 induced antigen-specific salivary secretory IgA and serum IgG.	[3]
	SCPAw alone or SCPAw-CTX	C5a peptidase loss-of-function mutants SCPAw	Cholera toxin (CT)	Animal study: mice, NAS	Intranasal immunization with SCPA induced antibodies which are able to clear GAS from the oral-nasal mucosa and lung.	[4,5]
Fibronectin-binding Protein	1) LCP-J14-FNBR-B<br>2) LCP-P25-FNBR-BT<br>3) LCP-P25-J14	J14 and/or FNBR-B or FNBR-BT	LCP & BPPCysMPEG	Animal study: mice, NAS	LCP-1 stimulated strong J14-specific cellular immune responses.	[6,7]
	1) LCP-J8-FNBR<br>2) LCP-J8<br>3) LCP-FNBR	J8 and/or FNBR which contain B and T-cell epitope	LCP & MALP-2	Animal study: mice, NAS	Better protective immunity by a dual antigen vaccine candidate against GAS	[8]

	SfbI-CTB	SfbI protein	CTB	Animal study: mice, NAS; & mouse skin infection model, SC	Anti-SfbI immunity: Effective against pharyngeal colonization. Inadequate for protection against systemic infections after skin colonization.	[9]
	FBP54-CT	FBP54	CT	Animal study: mice, SC, NAS or OR	High salivary levels of IgA antibodies were detected after oral and nasal immunizations. Mice subcutaneously or orally immunized with FBP54 survived significantly longer following GAS challenge	[10]
	FbaA-FA	FbaA	Freund's adjuvant (FA)	Animal study: mice, SC	FbaA has similar ability to M protein in inducing protective immunity against GAS challenge in mice	[11]
Serum Opacity Factor (SOF)	1) SOF-SfbI-CTB<br>2) SOF	SOF, SfbI	CTB	Animal study: mice, NAS	Immunization with SOF-SfbI stimulated strong systemic and mucosal immune responses against both antigens<br>Vaccinated with SOF alone were not protected against a mucosal challenge	[12]
Streptococcal Pyrogenic Exotoxins (Spe)	SpeB (extracellular cysteine protease)	A highly conserved extracellular cysteine protease SpeB	-	Animal study: mice, SC, IP	The SpeB vaccine induced non type-specific immunity to challenge with heterologous GAS.	[13]

	SpeC	Y15A/N38D or Y15A/H35A/N38D SpeC mutant	-	Animal study: Rabbit, SC, IP	These mutants were highly immunogenic but nonmitogenic for rabbit splenocytes and human PBMCs<br>These mutants may be useful as toxoid vaccine candidates	[14]
IL-8 Protease (SpyCEP)	J8-DT plus S2-DT combination vaccine	J8 & SpyCEP epitopes (S1-S6)	Alum	Animal study: mice, SC	The combination induces complete protection against hypervirulent GAS mutant CovR/S	[15]
Streptococcal Secreted Esterase (Sse)	Sse	Recombinant protein Sse	Alum	Animal study: mice, SC; mouse skin infection model, SC	Immunization with Sse induced protection against subcutaneous infection by more than one GAS serotype.<br>Anti-Sse antibodies are not opsonic<br>Anti-Sse antibodies cannot control systemic GAS infection.	[16]
Protein G-related Alpha2-macroglobulin Binding Protein (GRAB) & Metal Transporter of Streptococcus (MtsA)	1) EKL24-KLH<br>2) EIN19-KLH	EKL24, EIN19	Keyhole lymphocyanin (KLH)	Animal study: mice, SC;	Conjugates did not induce opsonic immune responses.	[17]
Superoxide Dismutase (SOD)	SodA	Recombinant SodA	-	Animal study: mice, SC mouse skin infection model, SC	Immunised mice produce high antibody titers that can opsonize GAS but did not offer protection during challenge.	[18]

Streptococal Inhibitor of Complement (SIC)	-	SIC	-	Identified only	SIC interferes with coagulation and fibrinolysis and thereby enhances bacterial survival	[19]
Extracellular Streptodornase D (SdaD)	-	SdaD and its homologue Sda1	-	Identified only	A natural mutation which led to longer Sda1 and SdaD was proved to confer increased activity on the protein.	[20,21]
Streptolysin O (SLO)	Combination vaccine SLO-SpyCEP-SCPA-ADI-TF	SLO; SpyCEP; SCPA; Arginine deiminase (ADI); Trigger factor (TF)	Alum	Animal Study: mice, IM	This combination vaccine induced high-titer antigen-specific antibody responses with bactericidal activity	[22]

IM: intramuscular; NAS: intranasal; SC: subcutaneous; IP: intraperitoneal; OR: oral

References

1. Sabharwal, H.; Michon, F.; Nelson, D.; Dong, W.; Fuchs, K.; Manjarrez, R.C.; Sarkar, A.; Uitz, C.; Viteri-Jackson, A.; Suarez, R.S., et al. Group A Streptococcus (GAS) carbohydrate as an immunogen for protection against GAS infection. *Journal of Infectious Diseases* **2006**, *193*, 129-135, doi:10.1086/498618.
2. Kabanova, A.; Margarit, I.; Berti, F.; Romano, M.R.; Grandi, G.; Bensi, G.; Chiarot, E.; Proietti, D.; Swennen, E.; Cappelletti, E., et al. Evaluation of a Group A Streptococcus synthetic oligosaccharide as vaccine candidate. *Vaccine* **2010**, *29*, 104-114, doi:10.1016/j.vaccine.2010.09.018.
3. Ji, Y.; Carlson, B.; Kondagunta, A.; Cleary, P.P. Intranasal immunization with C5a peptidase prevents nasopharyngeal colonization of mice by the Group A Streptococcus. *Infection and Immunity* **1997**, *65*, 2080-2087.
4. Park, H.S.; Cleary, P.P. Active and passive intranasal immunizations with streptococcal surface protein C5a peptidase prevent infection of murine nasal mucosa-associated lymphoid tissue, a functional homologue of human tonsils. *Infection and Immunity* **2005**, *73*, 7878-7886, doi:10.1128/IAI.73.12.7878-7886.2005.
5. Cleary, P.P.; Matsuka, Y.V.; Huynh, T.; Lam, H.; Olmsted, S.B. Immunization with C5a peptidase from either Group A or B Streptococci enhances clearance of Group A Streptococci from intranasally infected mice. *Vaccine* **2004**, *22*, 4332-4341, doi:10.1016/j.vaccine.2004.04.030.
6. Skwarczynski, M.; Parhiz, B.H.; Soltani, F.; Srinivasan, S.; Kamaruzaman, K.A.; Lin, I.-C.; Toth, I. Lipid peptide core nanoparticles as multivalent vaccine candidates against *Streptococcus pyogenes*. *Aust. J. Chem.* **2012**, *65*, 35-39, doi:10.1071/CH11292.
7. Schulze, K.; Ebensen, T.; Chandrudu, S.; Skwarczynski, M.; Toth, I.; Olive, C.; Guzman, C.A. Bivalent mucosal peptide vaccines administered using the LCP carrier system stimulate protective immune responses against *Streptococcus pyogenes* infection. *Nanomedicine: Nanotechnology Biology and Medicine* **2017**, *13*, 2463-2474, doi:10.1016/j.nano.2017.08.015.
8. Olive, C.; Schulze, K.; Sun, H.K.; Ebensen, T.; Horvath, A.; Toth, I.; Guzman, C.A. Enhanced protection against *Streptococcus pyogenes* infection by intranasal vaccination with a dual antigen component M protein/SfbI lipid core peptide vaccine formulation. *Vaccine* **2007**, *25*, 1789-1797, doi:10.1016/j.vaccine.2006.11.031.

9. McArthur, J.; Medina, E.; Mueller, A.; Chin, J.; Currie, B.J.; Sriprakash, K.S.; Talay, S.R.; Chhatwal, G.S.; Walker, M.J. Intranasal vaccination with streptococcal fibronectin binding protein Sfb1 fails to prevent growth and dissemination of Streptococcus pyogenes in a murine skin infection model. *Infect Immun* **2004**, *72*, 7342-7345, doi:10.1128/IAI.72.12.7342-7345.2004.
10. Kawabata, S.; Kunitomo, E.; Terao, Y.; Nakagawa, I.; Kikuchi, K.; Totsuka, K.; Hamada, S. Systemic and mucosal immunizations with fibronectin-binding protein FBP54 induce protective immune responses against *Streptococcus pyogenes* challenge in mice. *Infection and Immunity* **2001**, *69*, 924-930, doi:10.1128/IAI.69.2.924-930.2001.
11. Ma, C.Q.; Li, C.H.; Wang, X.R.; Zeng, R.H.; Yin, X.L.; Feng, H.D.; Wei, L. Similar ability of FbaA with M protein to elicit protective immunity against Group A Streptococcus challenge in mice. *Cell Mol Immunol* **2009**, *6*, 73-77, doi:10.1038/cmi.2009.10.
12. Schulze, K.; Medina, E.; Guzman, C.A. Intranasal immunization with serum opacity factor (SOF) of *Streptococcus pyogenes* fails to protect mice against lethal mucosal challenge with a heterologous strain. *Vaccine* **2006**, *24*, 1446-1450, doi:10.1016/j.vaccine.2005.06.039.
13. Kapur, V.; Maffei, J.T.; Greer, R.S.; Li, L.L.; Adams, G.J.; Musser, J.M. Vaccination with streptococcal extracellular cysteine protease (interleukin-1 beta convertase) protects mice against challenge with heterologous Group A Streptococci. *Microb Pathog* **1994**, *16*, 443-450, doi:10.1006/mpat.1994.1044.
14. McCormick, J.K.; Tripp, T.J.; Olmsted, S.B.; Matsuka, Y.V.; Gahr, P.J.; Ohlendorf, D.H.; Schlievert, P.M. Development of *Streptococcal pyrogenic* exotoxin c vaccine toxoids that are protective in the rabbit model of toxic shock syndrome. *The Journal of Immunology* **2000**, *165*, 2306-2312, doi:10.4049/jimmunol.165.4.2306.
15. Shet, A.; Kaplan, E.L.; Johnson, D.R.; Cleary, P.P. Immune response to Group A Streptococcal C5a peptidase in children: implications for vaccine development. *Journal of Infectious Diseases* **2003**, *188*, 809-817, doi:10.1086/377700.
16. Liu, M.; Zhu, H.; Zhang, J.; Lei, B. Active and passive immunizations with the streptococcal esterase sse protect mice against subcutaneous infection with Group A Streptococci. *Infection and Immunity* **2007**, *75*, 3651-3657, doi:10.1128/iai.00038-07.
17. McMillan, D.J.; Batzloff, M.R.; Browning, C.L.; Davies, M.R.; Good, M.F.; Sriprakash, K.S.; Janulczyk, R.; Rasmussen, M. Identification and assessment of new vaccine candidates for Group A Streptococcal infections. *Vaccine* **2004**, *22*, 2783-2790, doi:10.1016/j.vaccine.2004.01.043.
18. McMillan, D.J.; Davies, M.R.; Good, M.F.; Sriprakash, K.S. Immune response to superoxide dismutase in Group A Streptococcal infection. *FEMS Immunol Med Microbiol* **2004**, *40*, 249-256, doi:10.1016/S0928-8244(04)00003-3.
19. Frick, I.M.; Shannon, O.; Neumann, A.; Karlsson, C.; Wikstrom, M.; Bjorck, L. Streptococcal inhibitor of complement (SIC) modulates fibrinolysis and enhances bacterial survival within fibrin clots. *J Biol Chem* **2018**, *293*, 13578-13591, doi:10.1074/jbc.RA118.001988.
20. Podbielski, A.; Zarges, I.; Flosdorff, A.; Weber-Heynemann, J. Molecular characterization of a major serotype M49 Group A Streptococcal DNase gene (sdaD). *Infection and immunity* **1996**, *64*, 5349-5356.
21. Aziz, R.K.; Ismail, S.A.; Park, H.-W.; Kotb, M. Post-proteomic identification of a novel phage-encoded streptodornase, Sda1, in invasive M1T1 *Streptococcus pyogenes*. *Molecular Microbiology* **2004**, *54*, 184-197, doi:10.1111/j.1365-2958.2004.04255.x.
22. Timmer, A.M.; Timmer, J.C.; Pence, M.A.; Hsu, L.-C.; Ghochani, M.; Frey, T.G.; Karin, M.; Salvesen, G.S.; Nizet, V. Streptolysin O promotes Group A Streptococcus immune evasion by accelerated macrophage apoptosis. *J Biol Chem* **2009**, *284*, 862-871, doi:10.1074/jbc.M804632200.
